# New circumscriptions add two northern Andean species to *Kohleria* (Gesneriaceae)

**DOI:** 10.3897/phytokeys.179.65990

**Published:** 2021-07-12

**Authors:** John L. Clark, Lou Jost

**Affiliations:** 1 Science Department, The Lawrenceville School, Lawrenceville, NJ 08648, USA The Lawrenceville School Lawrenceville United States of America; 2 Lou Jost, Fundacion EcoMinga, Baños, Tungurahua, Ecuador Fundacion EcoMinga Ba&ntilde;os Ecuador

**Keywords:** Colombia, Ecuador, Gesneriaceae, *
Kohleria
*, taxonomy

## Abstract

Recent studies of type specimens and exploratory research expeditions in the northern Andes have resulted in an updated circumscription and recognition for two species of *Kohleria* (Gesneriaceae) in Ecuador and Colombia. A change in the rank from a variety to species is recognized for *Kohleria anisophylla* (Fritsch) Wiehler. The combination *Kohleria andina* (Fritsch) J.L. Clark & Jost, **comb. nov.** is provided here and a lectotype is designated. The updated circumscriptions of these two species are supported by morphology and geographic distribution. The presence of an epiphytic habit for *Kohleria* is discussed. Field images based on recent expeditions are provided to support the circumscriptions presented here.

## Abstract (es)

Los estudios recientes de las colecciones tipo y las expediciones exploratorias en el norte de los Andes han dado como resultado la actualización en la circunscripción y reconocimiento de dos especies de *Kohleria* (Gesneriaceae) en Ecuador y Colombia. Se reconoce el cambio de rango de variedad a especie para *Kohleria anisophylla* (Fritsch) Wiehler. Se presenta la nueva combinación *Kohleria andina* (Fritsch) J.L. Clark & Jost, **comb. nov.** con la designación de su lectotipo. La circunscripción actualizada de estas dos especies está soportada por caracteres morfológicos y distribución geográfica. Se discute la presencia del hábito epífito en *Kohleria*. Se presentan imágenes obtenidas en las expediciones de campo para soportar las circunscripciones propuestas aquí.

## Introduction

The flowering plant family Gesneriaceae, with over 3400 species and 150+ genera (Weber 2004; Weber et al. 2013) is in the order Lamiales. The family is divided into three subfamilies and seven tribes (Weber et al. 2013, 2020). The majority of New World members are in the subfamily Gesnerioideae and are represented by 1200+ species and 77 genera (Clark et al. 2020). The New World subfamily Sanangoideae is limited to one genus and one species (Weber et al. 2013, 2020). *Kohleria* Regel is classified in the tribe Gesnerieae Dumort. and subtribe Gloxiniinae G. Don (Weber et al. 2013, 2020).

*Kohleria* was monographed by Kvist and Skog (1992), who recognized 17 species. An additional two species were transferred to *Kohleria* from *Capanea* Decaisne ex Planchon (Roalson et al. 2005a) based on molecular phylogenetic analyses of the tribe Gloxinieae (Roalson et al. 2005b). Clark and Skog (2008) described *Kohleria hypertrichosa* J.L. Clark & Skog from the western Andes of northern Ecuador. The recognition of two more *Kohleria* species here brings the total number in the genus to 22 species.

Kvist and Skog (1992) broadly defined many species in their monographic revision of *Kohleria*. For example, Kohleria hirsuta (Kunth) Regel var. hirsuta (sensu Kvist and Skog 1992) includes more than 40 names representing 15 heterotypic synonyms. Kvist and Skog (1992) noted a wide range of morphological variation when circumscribing taxa, and that variation was attributed to hybridization. Molecular tools, fieldwork, and ready access to digital images are necessary for evaluating many of these broadly circumscribed species. A current doctoral dissertation project by Kimberly Hansen from Washington State University (USA) and an undergraduate thesis by Katherin Arango-Gómez from the Universidad del Valle (Colombia) are evaluating the phylogeny and taxonomy of *Kohleria* based on the use of molecular tools, herbarium specimens, and extensive field work. The updated circumscriptions provided here will hopefully play a role in facilitating these active projects.

## Results

### New generic placement requires new combination and lectotypification for *Kohleria andina*

#### Kohleria andina

Taxon classificationPlantaeLamialesGesneriaceae

(Fritsch) J.L. Clark & Jost comb. nov.

C651D84D-AF43-5148-AE25-6A5A8DA0BB0F

urn:lsid:ipni.org:names:77218228-1

Capanea andina Fritsch, Bot. Jahrb. Syst. 50: 431–432. 1913 (“1914”). Type: Ecuador. Andes Quitenses, Tunguragua, 1857, *R. Spruce 5178* (lectotype K000395097, designated here; isolectotypes: BM000953512, E00062367, G00370826, G00370838, K000395097).

##### Remarks.

One of the key characters discussed by Kvist and Skog (1992) as the basis for the generic circumscription of *Kohleria* was a terrestrial (i.e., non-epiphytic) habit. In the generic delimitation of *Kohleria* (Kvist and Skog 1992), the habit is described as herbs, subshrubs, shrubs, or rarely scandent shrubs. The terrestrial habit was considered a character by Kvist and Skog (1992) to differentiate *Kohleria* from closely related genera that are epiphytes or lianas. Phylogenetic studies by Roalson et al. (2005b) showed that *Kohleria* was paraphyletic with the exclusion of “*Capanea*”, a group of epiphytic subshrubs from the Andes. Thus, many of the features that differentiated “*Capanea*” from *Kohleria*, such as an epiphytic habit and four-valved capsules, are autapomorphic. The transfer of two species from “*Capanea*” to *Kohleria* is well-supported, and combinations were made by Roalson et al. (2005b). Roalson et al. (2005b) did not make a combination for *Kohleria andina* because it was considered a heterotypic synonym of *Kohleria affinis*. Examination of material in the field and in herbaria allowed us to recognize *K. affinis* and *K. andina* as different species. Outlined here are characters to differentiate *K. andina* from *K. affinis* (see Table 1 for a summary of the characters that are discussed below).

**Table 1.** Morphological differences and general distribution of *Kohleria affinis* and *K. andina*.

	*Kohleria affinis* (Fritsch) Roalson & Boggan	*Kohleria andina* (Fritsch) J.L. Clark & Jost
Corolla tube shape	usually narrow, rarely broad (Colombia)	broad
Corolla tube color	dark red to bright purple	white
Corolla tube trichome color	transparent	yellow
Corolla tube length	3–6 cm	< 3.5 cm
Peduncle and pedicel trichome color	transparent	purple
Distribution	widespread in Colombia, Ecuador, and northern Peru	endemic to the Ecuadorian province of Tungurahua)

**Figure 1.**
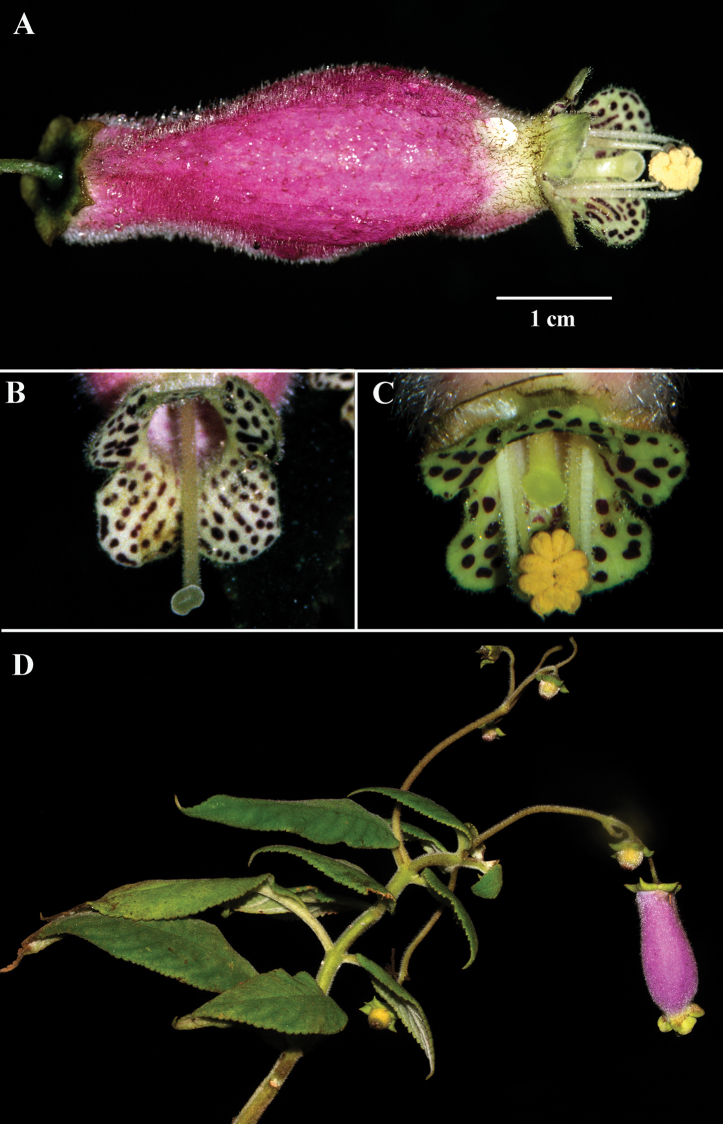
*Kohleria affinis* (Fritsch) Roalson & Boggan **A** lateral view of flower **B** female phase of mature flower **C** male phase of mature flower **D** habit (**A***Clark et al. 7698***B***Clark s.n*. **C***Clark et al. 12979***D***Clark et al. 15845*). Photos by J.L. Clark.

**Figure 2.**
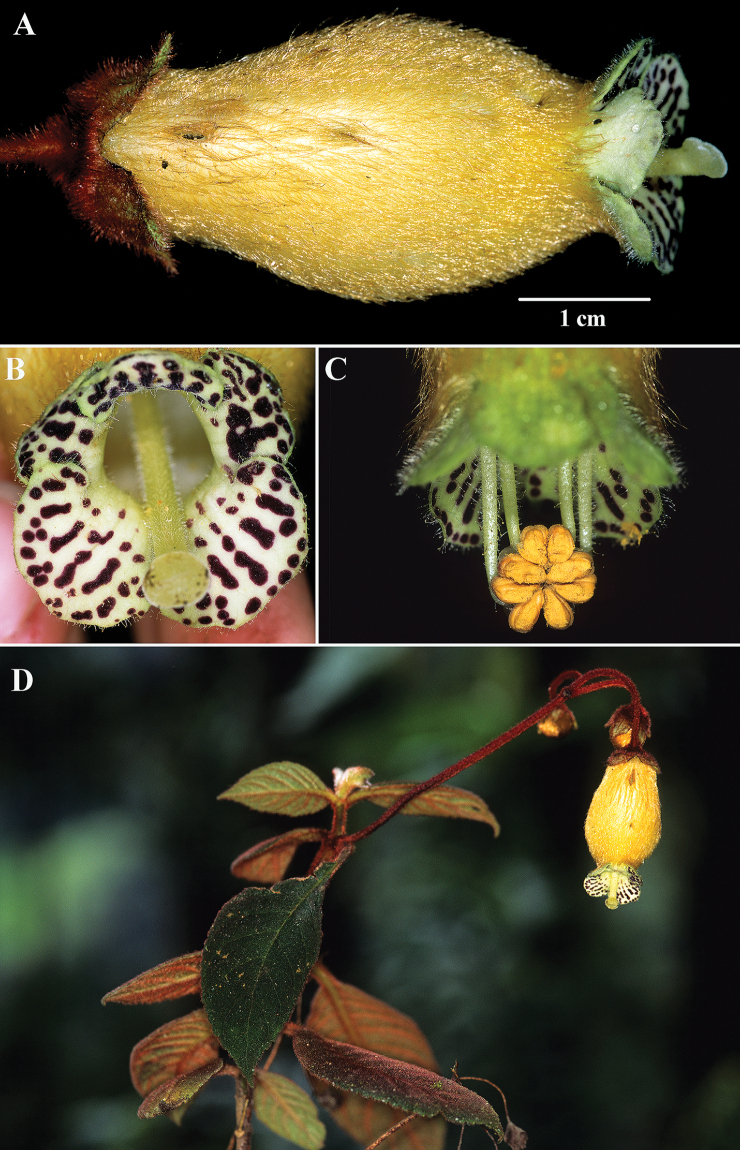
*Kohleria andina* (Fritsch) J.L. Clark & Jost **A** lateral view of flower **B** female phase of mature flower **C** male phase of mature flower **D** habit (**A–D***Clark et al. 7750*). Photos by J.L. Clark.

Another feature that defines the clade previously recognized as “*Capanea*” is the presence of resupinate flowers via a twisted pedicel. The androecium and gynoecium are located in the lower region of the corolla tube (Fig. 1B, C and Fig. 2B, C). In contrast, all other *Kohleria* and closely related genera have the androecium and gynoecium in the upper region of the corolla tube.

The flowers of *Kohleria affinis* are often photographed because of their conspicuous clusters of brightly colored purple-red corolla tubes with contrasting green lobes (Fig. 1). It is common to see individuals with 50+ pendent flowers, especially in abandoned cow pastures or recently cleared forests. Herbarium specimens do not preserve floral colors and most corollas dry uniformly black. Thus, corolla colors are challenging to determine on dried herbarium specimens unless noted by collectors in the descriptions. Use of field-based images, review of taxonomic literature, and examination of type specimens provided information for re-assessing the circumscription of *Kohleria affinis* and *K. andina*.

The corolla tube of *Kohleria andina* is white, but appears bright yellow from dense tomentose yellow trichomes (Fig. 2). In contrast, the corolla tube of *Kohleria affinis* is dark red to bright purple (Fig. 1). The corolla tube in most *Kohleria affinis* is narrow, but some populations from Colombia are broad. The corolla tube of *Kohleria andina* is consistently broad. Corolla length in *Kohleria affinis* is highly variable and ranges from 3 to 6 cm. In contrast, the corolla tubes of *Kohleria andina* are usually less than 3.5 cm long. Both species have bright green corolla lobes that contrast with dark purple spots on the inner surface (Figs 1, 2).

An additional character useful for differentiating *Kohleria andina* from *K. affinis* is the presence of dark red-purple trichomes on the peduncles and pedicels (Fig. 2A, D). The red-purple trichomes on the peduncles were noted by Fritsch (1913: page 432) in the protologue, “Pedunculi axillares elongati purpureo-villosi.” In contrast, the peduncles in *Kohleria affinis* are sparsely pilose and appear green due to transparent trichomes.

*Kohleria andina* and *K. affinis* are geographically separated by elevation. *K. andina* is endemic to elevations above 2500 meters on the western Andean slopes (Cordillera Oriental) in the Tungurahua province of Ecuador. In contrast, *Kohleria affinis* is widespread in the northern Andes of Colombia (Antioquia, Boyacá, Caldas, Caquetá, Cauca, Chocó, Cundimarca, Huila, Nariño, Putumayo, Quindío, Risaralda, and Valle del Cauca), Ecuador (Azuay, Bolívar, Carchi, Chimborazo, Cotopaxi, Esmeraldas, Imbabura, Loja, Napo, Pichincha, Santo Doingo, Tungurahua, and Zamora-Chinchipe), and northern Peru (Amazonas and Cajamarca). The authors’ field work from the upper slopes of Tungurahua, an active volcano in the western Andean slopes of the Cordillera Oriental, revealed little overlap. *Kohleria andina* is locally endemic to elevations above 2500 meters and *K. affinis* is widespread and located in forests below 2500 meters. Intermediate forms were not found here, indicating that these two forms are geographically separated by elevation and supported as different biological species.

##### Lectotypification.

Syntypes are from two distinct localities: *F.C. Lehmann 4869* (F0060498) from Colombia and *R. Spruce 5178* from K (K000395097) from Tungurahua, Ecuador. The specimen of *F.C. Lehmann 4869* (F) is more similar to the widespread *Kohleria affinis*. The specimen of *R. Spruce 5178* from (K) is similar to the locally endemic *Kohleria andina*, and is designated as the lectotype to stabilize this species concept. According to Fritsch (1913), Richard Spruce cites Tunguarahua as a locality and the specimens have characters that are congruent with the Tungurahua populations featured in the images here (Fig. 2). The lectotype has a corolla that is wide and more ampliate (Fig. 2) relative to the narrower corolla tube of *K. affinis* (Fig. 1). An additional character that is congruent with material from the type locality and the lectotype (*R. Spruce 5178*) is the presence of dark red trichomes on the peduncles and pedicels. In contrast, the peduncle and pedicel trichomes on *F.C. Lehmann 4869* are transparent and more similar to *K. affinis*.

### Revised species circumscription for *Kohleria anisophylla*

#### Kohleria anisophylla

Taxon classificationPlantaeLamialesGesneriaceae

(Fritsch) Wiehler

D3BAC67D-CB74-5A49-AA88-BB9A52AE8F15

Kohleria anisophylla (Fritsch) Wiehler.
Kohleria anisophylla (Fritsch) Wiehler, Selbyana 5: 62. 1978. Type: Based on Diastema anisophyllum Fritsch.
Kohleria villosa var. anisophylla (Fritsch) Kvist & Skog, Smithsonian Contr. Bot. 79: 70. 1992. Type: Based on Diastema anisophyllum Fritsch. Basionym.
Diastema anisophyllum Fritsch, Bot. Jahrb. Syst. 50: 408. 1913 (“1914”). Type: Colombia. [Nariño] Piedra Ancha, West of Andes of Tuquerres, *F.C. Lehmann 5843* (B, holotype not extant, lectotype K000509983, designated by Wiehler (1978: 62), isolectotype K000509984).
Nematanthus erianthus Bentham, Pl. Hartw: 231. 1846. Type: Ecuador. Pichincha: Quito towards Nanegal, *Hartweg s.n.* (holotype K000509985).
Columnea eriantha (Bentham) Hanstein, Linnaea 34: 391. 1865. Type: Based on Nematanthus erianthus Fritsch.
Diastema anisophyllum Fritsch var. quitense Fritsch. Bot. Jahrb. Syst. 50(4): 408. 1913 (“1914”). Type: Ecuador. [Pichincha] Quito, *W. Jameson s.n.* (holotype W).

##### Remarks.

**Figure 3.**
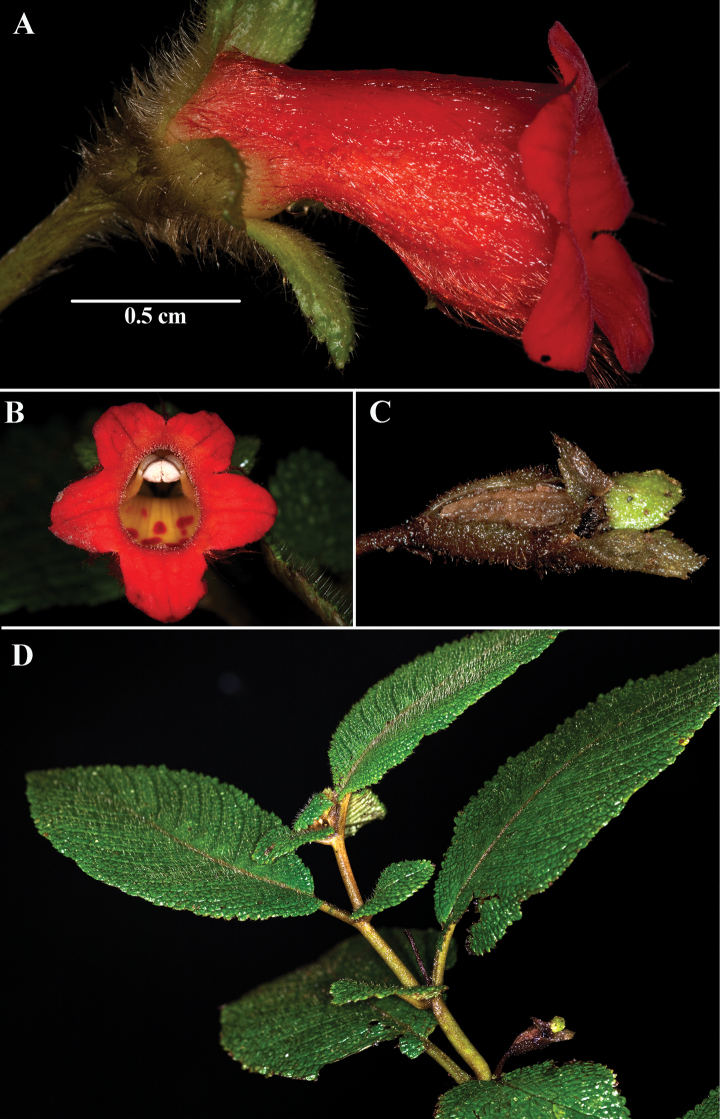
*Kohleria anisophylla* (Fritsch) Wiehler **A** lateral view of flower **B** front view of corolla **C** mature fruit **D** dorsiventral habit with anisophyllous leaves (**A, B***Clark et al. 10981***C***Clark 10948***D***Clark et al. 14295*). Photos by J.L. Clark.

**Figure 4.**
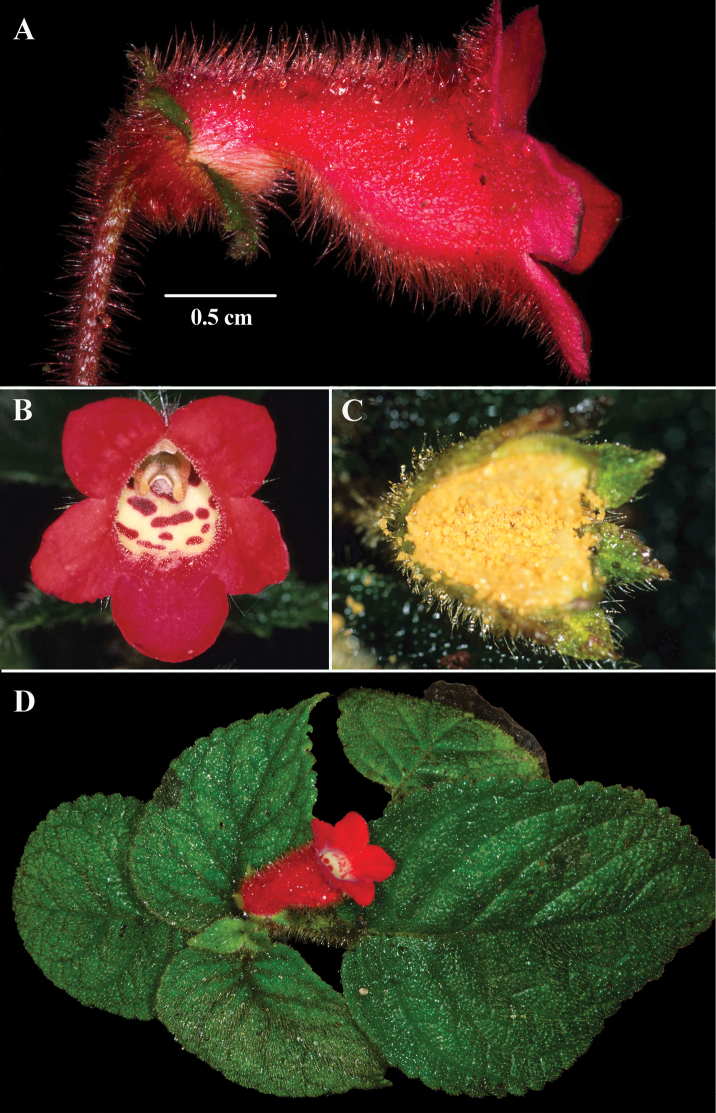
*Kohleria villosa* (Fritsch) Wiehler **A** lateral view of flower **B** front view of corolla **C** mature fruit **D** erect herbaceous habit with isophyllous leaves (**A***Clark et al. 14295***B***Clark 7331***C***Clark et al. 7400***D***Clark et al. 14295*). Photos by J.L. Clark.

**Figure 5.**
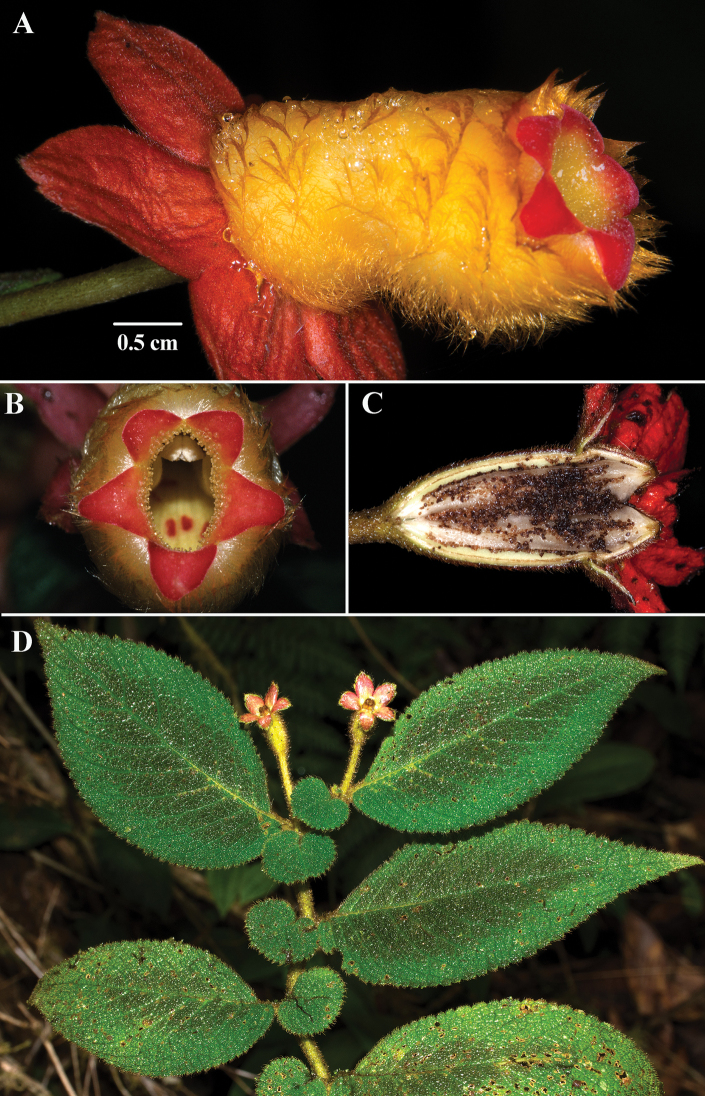
*Kohleria hypertrichosa* J.L. Clark & L.E. Skog **A** lateral view of flower **B** front view of corolla **C** mature fruit **D** dorsiventral habit with anisophyllous leaves (**A***Clark et al. 15900***B***Clark 6539***C***Clark et al. 10310***D***Clark et al. 14942*). Photos by J.L. Clark.

*Kohleria anisophylla* (Fig. 3) was previously recognized by Kvist and Skog (1992) as a variety of *Kohleria villosa* (Fig. 4). The strongly anisophyllous leaves and dorsiventral shoots (Fig. 3D) are more similar to *Kohleria hypertrichosa* (Fig. 5D) than *K. villosa* (Fig. 4D). All three species are found on the northwestern Andean slopes of Ecuador. Only *Kohleria anisophylla* is documented from Colombia (Nariño department). Wiehler (1978) made the combination *Kohleria anisophylla* and recognized it at the rank of species. Kvist and Skog (1992) recognized this taxon as Kohleria villosa var. anisophylla. Based on limited material, Wiehler (1978) cited the type (*F.C. Lehmann 5843*) and a recently collected specimen from Ecuador (*C. Luer & A. Hirtz 2672*). Kvist and Skog (1992) cited the same Ecuadorian collection and mentioned the study of eleven additional specimens. This species is common along the northwestern slopes of the Ecuadorian Andes, especially along the old road between Quito and Santo Domingo where many of the images were taken for Figure 3. Outlined here are characters to differentiate *K. anisophylla*, *K. villosa*, and *K. hypertrichosa* (see Table 2 for a comparison of characters that are discussed below).

**Table 2.** Morphological differences and general distribution of *Kohleria anisophylla*, *K. villosa*, and *K. hypertrichosa*.

	*Kohleria anisophylla* (Fritsch) Wiehler	*Kohleria villosa* (Fritsch) Wiehler	*Kohleria hypertrichosa* J.L. Clark & L.E. Skog
**Habit**	facultative epiphyte	terrestrial	facultative epiphyte
**Shoots**	dorsiventral	erect	dorsiventral
**Relative leaf size**	anisophyllous	isophyllous	anisophyllous
**Corolla vestiture**	villous	villous	tomentose
**Distribution**	Ecuador (Bolívar, Carchi, Pichincha) and Colombia (Nariño)	Ecuador (Bolívar, Carchi, Cotopaxi, Esmeraldas, Imbabura, Pichincha)	northern Ecuador (Carchi and Esmeraldas)

The recent transfer (Roalson et al. 2005b) of *Kohleria affinis* and *K. tigridia* (Ohlend.) Roalson and Boggan represented an autapomorphic synapomorphy of epiphytism in traditionally recognized *Kohleria*. What is noteworthy about *Kohleria anisophylla* and *K. hypertrichosa* is their previously unreported epiphytic habits. Thus, the presence of an epiphytic habit in *K. anisophylla* and *K. hypertrichosa* could represent an additional independent origin of epiphytism in *Kohleria*. Several populations of *Kohleria anisophylla* were observed and documented with dorsiventral shoots, a feature that is common in facultative epiphytes in other Gesneriaceae genera. Many members of *Columnea* have strongly anisophyllous leaves – especially species that are facultative epiphytes with dorsiventral shoots. Other species of Gesneriaceae that are facultative epiphytes with dorsiventral shoots include *Cremosperma anisophylla* J.L. Clark & L.E. Skog, *Drymonia anisophylla* L.E. Skog & L.P. Kvist, and the majority of species in *Monopyle* Moritz ex Benth. and *Trichodrymonia* Oerst. Likewise, *Kohleria anisophylla* and *K. hypertrichosa* are facultative epiphytes with dorsiventral shoots and anisophyllous leaves. In contrast, *Kohleria villosa* is a terrestrial herb with isophyllous leaves (Fig. 4D).

The corollas of *Kohleria villosa* and *K. anisophylla* are villous (Figs 3, 4). The corollas of *Kohleria hypertrichosa* are densely tomentose (Fig. 5). The specific epiphyte, “*hypertrichosa*” refers to the abundance of trichomes, which is why it is commonly known in the horticultural community as “Chewbacca,” a reference to the Wookie (fictional character) in the movie Star Wars.

*Kohleria villosa* and *K. anisophylla* are easily recognized when sterile. The opposite leaves of *Kohleria anisophylla* are consistently unequal in size or anisophyllous (Fig. 3D). In contrast, the opposite leaves of *Kohleria villosa* are consistently equal in size or isophyllous (Fig. 4D). In addition, the dorsiventral shoots distinguishes *K. anisophylla* from the erect shoots of *K. villosus*.

## Supplementary Material

XML Treatment for Kohleria andina

XML Treatment for Kohleria anisophylla
